# Barriers to Equity in Cancer Survivorship Care: Perspectives of Cancer Survivors and System Stakeholders

**DOI:** 10.1177/23333936211006703

**Published:** 2021-04-14

**Authors:** Tracy L.O. Truant, Leah K. Lambert, Sally Thorne

**Affiliations:** 1 BC Cancer, Vancouver, Canada; 2 University of British Columbia, Vancouver, Canada

**Keywords:** cancer survivorship, equity, cancer care, survivorship, health equity, Canada, qualitative research, interpretive description

## Abstract

As more cancer patients survive into post-treatment, the challenge of managing their survivorship care is confronting health care systems globally. In striving to deliver high quality survivorship care, equity constitutes a particularly troublesome challenge. We analyzed accounts from both cancer survivors and stakeholders within care system management to uncover insights with respect to barriers to equitable cancer survivorship services. Beyond the social determinants of health that shape inequities across all of our systems, the cancer care system involves a pattern of prioritizing biomedicine, evidence-based options, and care standardization. We learned that these lead to system rigidities that not only compromise the individualization essential to person-centered care but also obscure the attention to group differences that becomes indispensable to responsiveness to inequities. On the basis of these insights, we reflect on what may be required to begin to redress the current and projected inequities with respect to access to appropriate cancer survivorship supports and services.

## Introduction

Major advances in cancer treatment over recent decades have resulted in an exponential rise in the number of cancer survivors post treatment who will continue to experience the effects of cancer or its treatment on an ongoing or even permanent basis (Alfano et al., 2019; Canadian Cancer Society, Statistics Canada, the Public Health Agency of Canada, 2019). According to the Canadian Cancer Research Alliance (2017), across all tumor sites, two-thirds of Canadians diagnosed today will become long-term cancer survivors. For the most part, the nature and complexity of the long-term repercussions these cancer survivors face mean that the primary point of reference for their care is not the traditional oncology system, but rather their care must be transitioned to primary care systems or new forms of specialized survivorship services.

Within the context of these newer systems and models of care, there is increasing concern about the problem of equity, specifically that there are certain populations whose needs will not be met as well as others (Alfano et al., 2019; Caron et al., 2018; CPAC, 2016; Hastert et al., 2019; Horrill et al., 2019; Kano et al., 2020; Keesing et al., 2015; Shapiro, 2018). In the Canadian context, where universal access to health care is a fundamental core principle, it is well recognized that the same commitment to equity that assures us that all patients will have equal access to high quality cancer treatment is not well delineated or assured at the level of survivorship care (Easley et al., 2016; Truant et al., 2016). In contrast to a high level of attention to cancer detection and treatment, the period after active cancer treatment has been “largely neglected in advocacy, clinical practice, and research” (CCRA, 2017, p. 7) In fact, the idea that vast numbers of cancer survivors might have significant residual needs beyond that which could be provided by the formal oncology system went largely unrecognized until the US Institute of Medicine’s seminal report in 2006 (Hewitt et al., 2006). Prior to that time, for the most part, both care providers and society at large had assumed that patients who survived their treatment and did not have evident disease recurrence were “cured,” and expected to “get back to normal.”

While systems have begun to address this gap in the years subsequent to that landmark 2006 paper (Mollica et al., 2020), we know that post-treatment care for many cancer patients continues to fall between the cracks (Alfano et al., 2019; CCRA, 2017; CPAC, 2018). There are many reasons for this, including the complexity and diversity of potential long-term and late effects from cancer and its increasingly complex treatments; the number of cancer cases, including metastatic cancers, that are now being understood as chronic conditions; and the variety of symptoms, biomarkers, and comorbidities that patients may attend to in their own efforts to monitor their health and ensure early detection of any recurrence (Mayer et al., 2017; Truant et al., 2016). Where effectiveness of cancer survivorship care systems has been evaluated, outcomes from a patient perspective have rarely been included in these assessments (Birken et al., 2018). These are not core competencies readily available to the general practice community, and in fact reflect a fairly specialized form of practice within which there are many unknowns and evolving knowns (Easley et al., 2016; Shapiro et al, 2016). From prior work (Truant, 2018; Truant et al., 2019), we know that some cancer patients require high levels of psychosocial support due to the vulnerability associated with having had to confront their mortality with the diagnosis of a potentially lethal disease. Others require physical and occupational rehabilitative support, or support to build new lifestyle options to replace those that are no longer possible or available to them. Many people require rapid responsiveness to new and/or unfamiliar symptoms, help interpreting whether their concerns are or are not cancer related, and evolving patterns of surveillance as evidence-based, best practices evolve. Because of this complexity across contexts, conditions, and circumstances, the delivery of survivorship care must be personalized and individualized (Alfano et al., 2019), However, where survivorship practice guidelines exist, for the most part they are based upon expert consensus rather than evidence (Shapiro, 2018).

Because of the enormity and complexity of the need, various models of cancer survivorship care have emerged across Canadian jurisdictions in an attempt to address this growing concern. Despite considerable progress since the landmark Hewitt et al. (2006) paper raised the alarm, a recent review in the USA concluded, “There are still those who survive their cancers but are lost in transition, who do not get the care they need, who find the health care system confusing and uncoordinated, and who continue to suffer with and die of the late and long-term effects of curative cancer treatments” (Nekhyludov et al., 2017, p. 1980). Similarly, on the basis of a Canadian study of cancer survivorship experiences, it was found that “Many were uncertain of who was in charge of their care or who they should contact with cancer-related questions, particularly as they transitioned from acute care to the survivorship phase. In some cases, the patients were expected to be the managers of their care whether they wanted to be or not” (Easley et al., 2016, p. 825). Thus, it remains apparent that communication and coordination between survivors, providers and care sectors through this phase of the cancer journey is highly problematic and the current situation, characterized by relatively ad hoc systems of cancer survivorship service, is clearly both inequitable (Alfano et al., 2019) and unsustainable (Mayer et al., 2017).

In this study, we contributed to an understanding of what an equitable high quality cancer survivorship care system might look like in the Canadian context through a qualitative exploration of the perceptions of both cancer survivors and cancer survivorship system stakeholders as to what the major equity barriers entailed and how they might best be addressed.

## Methods

Drawing on Interpretive Description as a qualitative inquiry approach designed for the applied knowledge needs of the practice disciplines (Thorne, 2016), the primary source of data for this study was interviews focused on soliciting understandings from two distinct perspectives—that of a diversity of cancer survivors, and that of key informants in strategic positions within the cancer survivorship care delivery system. Interviews with these two groups of participants focused on obtaining an understanding of how structural and contextual factors (e.g., social, political, economic, and/or personal) might layer and intersect to influence survivors’ access to and experience of equitable high quality cancer survivorship care.

Using purposeful maximal variation sampling, a range of cancer survivor participants were recruited, explicitly focusing on diversities with respect to age, ethnicity, type of cancer, time since completion of primary treatment, cure/palliative/long-term or metastatic status, rural/urban location, immigration status, socioeconomic status, use of complementary and integrative therapies, and experience of late symptom and/or side effect trajectories. To increase the opportunity to reach those who might experience the greatest inequities, we explicitly looked for those who might be outliers within the system, such as those with intersecting challenges, rare cancers, or complicating circumstances. All were 18 years of age or above, diagnosed as an adult, and had completed primary cancer treatment in the province of British Columbia. Exclusion criteria included inability to communicate in English and non-melanoma skin cancer.

A total of 34 survivor participants reflecting a wide range of equity groups and circumstances (26 female, 8 male; ranging in age from 24 to late 80s; most being between ages 51 and 70; with a majority representing breast [15] and hematological [10] primary sites) were recruited using hard copy and electronic posters and fliers in public and private spaces in various locations around the province of British Columbia, Canada, including both urban and rural/remote regions. We found it difficult to enumerate their equity-related conditions in any systematic manner, as these were individuals, living lives shaped by multiple and intersecting factors, and many resisted categorization into neat and tidy social determinants of health groupings. Using a semi-structured, open-ended interview guide, we explored their experiences of engaging with survivorship care, including barriers to access, their perceptions on the degree to which their health needs were being met, and their insights as to how to design survivorship care resources that were both high quality and equitable. Ethics approval was obtained from the local university review board (UBC BREB #H -14-0382) and informed consent was obtained in writing prior to each interview. Interviews were face-to-face or by telephone according to participant preference and audio recorded (most lasted 60–90 minutes). All interviews were transcribed verbatim using non-identifying signifiers for confidentiality and all data was stored in a secure location. While it was clearly recognized that the accounts of 34 survivors could not capture the full spectrum of diversities that could potentially contribute to barriers to equity, the sample included sufficient richness and variety of cancer stories to allow for analysis of relevant themes and to surface original insights.

In addition to the survivor interviews, we purposefully sampled 12 key stakeholders (6 physicians, 6 nurses; 11 female, 1 male) holding leadership roles across a range of survivorship and cancer care programs, services and resources, and representing various communities within of our own province (7), other provinces (4), or national organizations (1). These interviews provided insight into the current and desired state of cancer survivorship care, as well as recommendations from their perspectives on how to minimize disparities and improve equitable access to high quality cancer survivorship care for all, despite inevitable fiscal constraints.

Data analysis involved repeated close reading of the interview data using constant comparative analysis to discern patterns and themes related to the focal question driving the study. In this manner, we oriented our analytic lens toward insights on factors that explained inequities from their diverse perspectives and recommendations on what would constitute an equitable high quality system of care for survivors. The report that follows is an interpretive synthesis of key themes across the two data sets of interviews that reflect perceptions of how these barriers work and how we might begin to address them.

## Findings

Our findings with respect to the equity gap in cancer care survivorship are captured in two broad themes that were discernable across the data from these various perspectives. First, we report on conditions within health care systems that serve as barriers to distributive justice, and second, we consider what survivors and system stakeholders believe needs to change in order for our health care systems to begin to address survivorship care in an equitable manner.

### How Barriers to Distributive Justice Widen the Equity Gap

Much has been written about barriers to high quality survivorship care as the enormity of the challenge is being felt across health care systems. As expected, we found clear evidence of those same barriers in the accounts of both survivors and system stakeholders. However, we were also able to dig deeper into their experiences and perspectives to investigate why those barriers might be so inequitable. Our findings therefore focus on that aspect of the in-depth narrative accounts, represented here in the form of two pervasive and intersecting conditions within health care systems that make adaptations to meet the changing needs of patient populations so challenging. We depict these conditions as a culture of privileging a biomedical perspective within care system design and resource allocation, and an attitude of “institutional arrogance” that creates blindspots with respect to patient/survivor experience that renders systems inflexible.

#### Prioritizing the biomedical lens

While cancer patients who have completed primary treatment commonly look back on their care within the active treatment phase as being somewhat holistic, most experience a sharp reduction in consideration of follow-up service beyond ongoing biomedical care. As one stakeholder explained,

*When the treatment’s over and, ‘Hey, here you are, go back to your good life,’ individuals feel like we have dropped them off the edge of a precipice, to say, ‘Okay, you survived now, you carry on with your life,’ without any kind of ways of helping them through that transition.*

In particular, we heard that, despite continued high levels of psychosocial need, many survivors experienced major gaps in that aspect on completion of their treatment. As one expressed it, “If there is a cancer survivorship system I would really like to know about it.” This individual had experienced “tough times” following the completion of treatment, but felt there was no one available to discuss those concerns. Another described trying to rationalize why this was the case.

*As a patient, you sort of say, ‘Get over yourself. They’re treating your body. They’re not treating your spirit, but they’re here to treat your body.’ But you know, I see them all as being interrelated. I don’t see how you can, you know, separate them out.*

Further, we heard that this abdication of care responsibilities once active medical management had concluded could be differentially interpreted by various communities. As one stakeholder explained,

*Unfortunately, I think that Indigenous peoples in Canada understand survivorship probably more than any other one group in Canada. . . So when you say, well, you’re cancer-free for five years, or the treatment is over, the chemotherapy is done, the radiation is completed, or you’re waking up in the recovery room and you’re hearing this voice saying that the surgery is over, you realize that that’s just one point in time, and nothing is over.*

For the few who were able to access wellness-focused services, there was a sense that these were limited to such modalities as meditation, relaxation, or physiotherapy, and that there were few services available with respect to individualized psychosocial care. Rather, much of what was available was population-based and tailored to needs that were assumed to be common, and access depended entirely on geographical locality. A stakeholder explained the challenge,

*So, I just think we need to figure out not one model because one size isn’t going to fit all, and, you know, making it appropriate for people, irrespective of where they live and who they are and what their socioeconomic status is and whether they’re from multicultural groups, all the rest of that really. . .a diversity and creating something that is not only centred around that individual, but is—works from within that individual that will really drive some change.*

Study participants observed that what was actually available seemed contingent on who was working in each region and what services they felt inclined to offer. In this manner, while individual champions for a supportive modality such as meditation seemed to have found ways to set up programs in which they were interested, there was no apparent consideration of comprehensive or coordinated services that might address a wider range of unmet needs.

Study participants also observed that, where larger survivorship programs and plans existed, the foundation always reflected a biomedical orientation, with broader needs such as psychosocial support coming in last as an afterthought in the plan of care. One system stakeholder interpreted this as being entirely opposite to the ideal of being “patient centered,”

*Well, the first thing is wipe out all those survivorship plans immediately, number one, and I’ll tell you why. Because when you work with patients and family members, design what it’s going to be, you’re going to get greater uptake. And I still would suggest that those survivorship plans are based on a medical model, and definitely not on person-centred in any way, shape or form.*

Further, there was no consistent perception among the survivors we interviewed that they actually did have a survivorship plan in place of any kind. As one survivor explained,

*To me, the healthcare system isn’t a system. To me the healthcare system is a bunch of components that operate pretty much in isolation. Well, not totally, but you know, they’re not as interconnected as they should be. You know, that your health information, your health records, the communication between various providers, is really poor. You know, there are a lot of gaps and things fall through the cracks during transitions from one system— you know, one area of the healthcare system to another. Information doesn’t get passed on. And that was one of the biggest things that complicated. . .my journey.*

This view that survivorship programming, where it existed at all, focused on biomedical rather than other aspects that are important and meaningful to survivors, was also confirmed by stakeholder study participants.

*We still get so oriented in the medical system to say, oh, we should be watching for it [cancer] if it’s coming back, but we forget all those other factors around good secondary prevention, good health, for Pete’s sake, over all, right?*

In most instances, there was a perception that it took considerable effort and initiative for survivors to seek out and locate necessary services, and that much of what was available for them to find, privileged a certain demographic of patients over others. Beyond self-evident forms of privilege in access to services (e.g., financial resources, education, access to transportation, and mobility), participants also described those who were younger, those with breast cancer rather than other tumor sites, and those who had highly developed advocacy skills, such as through professional training, as being more likely to obtain survivorship care. Several of our study participants explained that since many survivorship services had originally come to exist as a result of breast cancer advocacy, that which was available was more tailored toward that population than the wider cancer survivorship context. Both survivors and stakeholders made it clear that many patients simply do not find their way to the kinds of services and supports they might desperately need, even when they are actively seeking these resources.

Another observation made by both survivors and stakeholders was that many of the supports and services that survivors valued and needed were unlikely to be available outside of the context of being part of a research study. Thus, they perceived eligibility to participate in clinical trials as being a significant equity driver, in that registering in a trial was often a ticket into higher quality concurrent supports. Since trials tend to privilege the dominant majority population in their inclusion criteria, this too became a barrier to equity. They also noted that in times of fiscal constraint, the services most likely to be discontinued—services such as nutrition, psychology, or physiotherapy—were predictably those considered less critically important by the biomedical community, regardless of patient perspective.

#### Normalizing institutional arrogance

The second, and often inter-related, pervasive condition to which many of these cancer survivors and stakeholders referred to when they reflected on why they thought systems were so slow in adapting to growing patient needs was a form of system blindspot or rigidity that one stakeholder referred to as “institutional arrogance.” The opinions and ideas within the accounts upon which this theme has been built had to do with a culture of efficiency, with the conviction that serving what was perceived to be the dominant majority was sufficient, and that an evidence-based and standardized service delivery model was the best approach to serve the greater good. From the survivor perspective, the contrast between attentiveness to the biomedical condition and unwillingness to respond to the resulting, consequential human experience was dramatic. As one survivor explained,

*It seemed like the more I tried to tell people, ‘I’m struggling emotionally. I don’t have anybody to talk to. I don’t have emotional support. I don’t have supports at home. I’m having a hard time eating anyway because of, you know, problems with my esophagus and stuff, and then I don’t have anybody to help me out, cook me meals, whatever, so if I don’t make myself something, I’m not getting anything.’ And it was kind of like, ‘Yeah, well, you’re done your treatment. Next.’ And I really felt like a number.*

As another said, “It’s so black and white with them. You have to follow a chart and it’s like, ‘Well, no, it’s been six weeks. You should be fine.’ Well, I’m not fine.”

Many survivors and stakeholders interpreted this imbalance as a misguided and short-sighted efficiency orientation throughout the health care system. As one stakeholder described,

*I’ve experienced that point in time in [our province’s] medical care history, when we went from a care model to a business model. That has been the downfall of our health care system ever since. So, I’m aware of all of that, but I still say right now to you, if you’re looking to save money, and that seems to be the top criteria these days of any new programs, this [survivorship care] would save money.*

Several stakeholders further noted that the care system was highly inflexible, making change extraordinarily challenging. As one expressed it:

*We talk about patient-focused, patient-focused, patient-focused, but it’s very difficult to do system change that’s patient-focused because the system is still systems-focused and it’s physician-focused and it’s funding-focused. So the flexibility that’s required to create meaningful change when you’re looking at models of care, for example, is very, very difficult.*

This rigidity and concern for cost constraint inevitably led those working within the system to focus on the priority tasks and develop blindspots with respect to unmet need; as one survivor put it “People are just working their butts off, and they forget that there’s a human sitting in the bed.” As a stakeholder confirmed, “I know nobody wants to hear this—we are looking at return on investment. When we do this, what are the outcomes? What are the outcomes for the individuals, what are the outcomes for the system, right?”

Both patients and stakeholders observed that, in a health care management culture that prioritized efficiency, treatment systems were becoming continually more narrow and less comprehensive, focusing on implementing what were seen as the basic requirements of service delivery rather than expanding to meet the more holistic needs. In the context of cancer care, this attitude prioritized active treatment and oncology specialist care over more comprehensive and chronic or ongoing support, as one stakeholder explained:

*In cancer care, as soon as the patient is no longer receiving the treatment in the cancer clinic and no longer being seen in the cancer clinic, there is no funding model to enable their care, right? There’s no money. It’s like you’re back to your primary provider and we’re not giving them any more money, good grief, to be able to do prevention, to be able to actually help you to live healthy, to work with you around identifying what some of those late and long-term effects are, and managing them. There’s no—there is no pocket of money to do that. It’s—the money is so based on, you know—you know this—the institutions, and not where it needed to be, which is focusing on health.*

As another stakeholder noted, this imbalance becomes particularly damaging in a climate in which the specialty oncology sector is permeated by an attitude of superiority over the primary care sector, inhibiting meaningful coordination between the two.

*Instead of creating trusting relationships and supporting quality care along with control, there’s this culture of institutional arrogance that goes along with that, you know, actively destroying the trust in and the relationship of the primary care provider and the patient. And you know, we have many. . . divisions of family practice who want to build capacity in all specialty areas, but particularly chronic diseases and the cancer part of that, and are saying to us, ‘You know, we want to care for these patients. Just tell us what we need to do. Give us the support, give us the access to expertise, give us the information. We will be accountable and responsible for that.’ And so we hear that loud and clear and we know that that needs to happen.*

As an example, they referenced the implementation of nurse practitioner roles within the local cancer care system, not to expand what the system could offer, but rather as a means by which to offload capacity from those who were seen as the core care providers—the oncologists. However, although these nurse practitioners were tasked with covering “primary care” functions that had been eating into the available clinical time of the oncologists, system leaders were also using this particular expansion to justify reducing staffing within other nursing designations and roles. Thus, for many patients, changes that were being defended as service expansion decisions actually resulted in service reductions.

*As a clinical nurse specialist there, what I felt I was doing more of was supporting people to manage the system, the healthcare—conventional healthcare system, and that was my job, really, to help people with their communication, with healthcare providers, managing the treatment. But for me, personally, I’d like to put my energy, creative energy into something other than propping up a system.*

### Envisioning Alternatives for Equitable High Quality Survivorship Care

Reflecting on these normalized attitudes of standardization, efficiency, and the primacy of specialty services, both survivor and stakeholder study participants had thoughts about what needed to change to begin to address survivorship needs in an equitable manner. For many, the key was the integrity of the human being at the center of care, as this survivor explained.

*And I think the young doctors that are coming up, in my mind, are the ones that are going to change the system that isn’t wrong, it’s just—it’s got a different focus. It’s less about the patient. It’s more about procedures and drugs, and they always need to remember that there’s a patient sitting in that bed. And when that begins to happen, I believe that healthcare is going to be more empathetic, you know, quantum healthcare, like, you know, this whole idea of integrating everything to make it all come together.*

Many recognized potential steps in the right direction by involving patients and survivors on committees responsible for the design of person-centered systems and programs, but also felt that this was tokenism due to the many layered and intersecting forces preventing them from fully informing these processes. As one stakeholder commented,

*How to close the gap between the professionals and the patients? Like, you have to close that gap because we are no different. We have expertise, but so do they. They’re experts on their experience, and we don’t let that expertise shape what we provide. And to me, that’s the starting place. . . I don’t know what it’s like to live with cancer. I’m fortunate. But I do know what it’s like to listen and try and shape a program based on what people are saying they need. You know, it’s so basic to me. So not the token, you know, patient on the committee. . .but they need to be linked into the system.*

Beyond steps to move closer to the capacity to individualize survivorship approaches, many reflected on the idea of distributive justice, or trying to distribute the burdens and benefits of available service across the full spectrum of potential service recipients. They felt that distributive justice was often lost within the ideology of scarcity and the management strategy of continually finding efficiencies. Survivors itemized numerous structural and technical barriers to self-advocacy that inevitably created inequities and could theoretically be readily addressed, as one seemingly minor barrier—a complaint line—seemed to exemplify.

*It’s not a person that answers the phone. So right there, they’re stopping you because they can then screen your call and see if they want to answer you or not. . . So if you want to do some proper self-advocacy, you have to do it on their terms. So you know, they really are setting up barriers for people who complain, if you want to put it that way.*

Stakeholders in particular wondered why, within a prevalent management rhetoric of constant quality improvement and system responsiveness, the most vulnerable and silent populations remained those with the most unmet needs. Some suspected that accountability structures that relied upon highly selective “reporting metrics” allowed many of the inequities associated with non-dominant groups to slide under the radar. As one stakeholder reflected on the particular gaps for Indigenous cancer survivors and those from remote localities:

*I think one of the most important things . . . is to recognize that you’re at a different starting point. And when you’re at a different starting point, you cannot treat everybody equal, or you will never get to that point of equity or equality that you’re aiming for.*

## Discussion

We recognize that this study is limited to a particular health care delivery system context and to a sample comprised of a fraction of the potential inequities that can and do arise in the survivorship care context. It is in the nature of studies focusing on health and system inequities that a comprehensive representation of all possible equity-seeking groups is inconceivable. We do feel that the survivors we recruited into this study contributed a reasonable range of conditions and experiences to allow us to consider the larger question of equity. We also believe that, by triangulating their accounts with the perspectives of system stakeholders concerned with the provision of equitable survivorship care, we have provided a window into the challenge that equity constitutes in this context. Therefore, given the high level of expressed concern worldwide with respect to gaps in cancer survivorship care and inadequate models for such care (Hastert et al., 2019; Mollica et al., 2020), we believe that the critically reflexive insights that this set of survivors and stakeholders has surfaced will be of relevance within our wider thinking.

It is clear from the accounts of both cancer survivors and system stakeholders in this study that cancer survivorship has not yet been sufficiently well positioned as a health system priority to allow for comprehensive systems of care, especially for those who may experience the highest unmet need. Placing the accounts obtained in the current study within the wider literature, it is apparent that quality and equity seem established deficits not only within existing cancer services but also within the wider health care systems in which they operate (Mayer et al., 2017; Truant et al., 2016). What these accounts add to this observation is an interpretive explanation for the complexity of these barriers if we are to aim toward a more ideal survivorship care context that accounts for the very real differences and inequities within actual experience (Caron et al., 2018; Horrill et al., 2019).

Among the concerns highlighted within these accounts are the paternalism of biomedicine and the managerialism of health care delivery systems. These are both prevalent themes in the wider dialog that critically reflects on the ideas that continue to shape modern health care (Bandini, 2020; Molina-Mulia et al., 2017). The mantra of evidence-based practice, which is so prevalent with the cancer care system, itself becomes a complicating factor with respect to equity (Alfano et al., 2019). In other words, the more unusual or distinctive the individual circumstances, the less likely they can and will be accommodated within the system. Further, the evidence ideology absolves the system from noticing or taking responsibility for the inequity in that it assumes the primacy of attending to the majority need (Nekhyludov et al., 2017). As our cancer survivorship care systems continue to evolve within an evidence-based mandate and increasingly quantified reporting structures, the rigidities within our systems may further exacerbate this imbalance, focusing resources and attention to that which serves a majority of cancer survivors and ignoring that which appears not to affect the reporting bottom line.

What seems missing within many of our systems are meaningful mechanisms to detect and respond to individuals and groups of cancer survivors who are not faring well, nor the perspectives of clinicians providing care. Thus, we do see promise in the increasing enthusiasm for patient reported outcomes, as well as for virtual mechanisms of health service delivery such as tele- and virtual health. We also see promise in the kinds of initiatives, such as Wellspring Canada, that legitimize the significant needs of those whose lives have been forever altered by having had cancer (Perry Brinkert & Valois, 2020; Santa Mina et al., 2017). Much of what such persons cope with is iatrogenic—secondary to disease or treatment—and much of it can be significantly eased with appropriate supports and resources. We are heartened by explicit statements in national cancer strategy documents such as the 2019 to 2029 Canadian Strategy for Cancer Control (Canadian Partnership Against Cancer [CPAC], 2019), that equitable access to high quality services, including survivorship services, is clearly recognized as a priority concern. We see significant potential for nursing to play an extensive role within the evolution of cancer survivorship approaches extending well beyond the existing silos of care. However, we also recognize that when biomedical priorities dictate who has direct access to nursing services, or how nurses are positioned or not positioned in our systems of care, we are not yet reaching those whose need may be greatest.

We cannot avoid concern over cost, but surely there must be more effective ways of considering it without it being the driver of all that we do. When efficiency models dominate over care quality considerations, we feel confident that inequities in service will be exacerbated, and our findings would certainly support that contention (Dean et al., 2018). Therefore, it is imperative that we develop mechanisms by which to demonstrate the cost-effectiveness in person-centered care and in meeting the unmet need of non-dominant population groups from the outset of the cancer care trajectory. Positioning our clinical research within the wider context of a critical reflection on societal health inequities becomes one such mechanism to sustain a focus on genuine quality. “Race, ethnicity, educational level, neighborhood, and job should not define either the care a person receives or the outcomes of care that are possible” (Horwitz, 2016, p. 1232). We know theoretically that upstream care—better screening, health promotion, supportive services—will ultimately reduce downstream morbidity, and we need the next generation of scholars to find better ways to capture that idea in a form that speaks strongly and effectively to an evidence-oriented health care policy and planning audience.

## Conclusion

Cancer survivorship presents our health care systems with an intriguing set of problems, not all of which are well contained within our existing cancer care delivery system ideologies and structures. As our various national health care systems grapple with the extraordinarily complex challenge that the increasing number of cancer survivors now poses, we believe that awareness of these kinds of structural and attitudinal barriers will be an essential component of ensuring both quality and equity. By providing an illumination of this challenge from the perspective of diverse cancer survivors and experts with a stake in systems that are as effective as possible in meeting the need, these findings offer the opportunity to look beyond the typical service delivery outcome measures and population-based statistics into the human lives that are so profoundly affected by cancer and to the wider societal forces that are so influential in shaping the role our existing systems have upon those lives.
